# External Beam Accelerated Partial Breast Irradiation Yields Favorable Outcomes in Patients with Prior Breast Augmentation

**DOI:** 10.3389/fonc.2014.00154

**Published:** 2014-06-19

**Authors:** Rachel Y. Lei, Charles E. Leonard, Kathryn T. Howell, Phyllis L. Henkenberns, Timothy K. Johnson, Tracy L. Hobart, Jane M. Kercher, Jodi L. Widner, Terese Kaske, Lora D. Barke, Dennis L. Carter

**Affiliations:** ^1^Radiation Oncology, Rocky Mountain Cancer Centers, Littleton, CO, USA; ^2^Radiation Oncology, Rocky Mountain Cancer Centers, Aurora, CO, USA; ^3^SurgOne, Littleton, CO, USA; ^4^Invision Sally Jobe Diagnostic Breast Center, Greenwood Village, CO, USA

**Keywords:** accelerated partial breast irradiation, breast augmentation mammoplasty, external beam radiotherapy, image-guided radiotherapy, intensity-modulated radiotherapy

## Abstract

**Purpose:** To report outcomes in breast cancer patients with prior breast augmentation treated with external beam accelerated partial breast irradiation (EB-APBI) utilizing intensity-modulated radiotherapy or 3-dimensional conformal radiotherapy, both with IGRT.

**Materials and Methods:** Sixteen stage 0/1 breast cancer patients with previous elective bilateral augmentation were treated post-lumpectomy on institutional EB-APBI trials (01185132 and 01185145 on clinicaltrials.gov). Patients received 38.5 Gy in 10 fractions over five consecutive days. Breast/chest wall pain and cosmesis were rated by patient; cosmesis was additionally evaluated by physician per RTOG criteria.

**Results:** The median follow-up from accelerated partial breast irradiation (APBI) completion was 23.9 months (range, 1.2–58.6). Little to no change in cosmesis or pain from baseline was reported. Cosmetic outcomes at last follow-up were judged by patients as excellent/good in 81.2% (13/16), and by physicians as excellent/good in 93.8% (15/16). Ten patients (62.5%) reported no breast/chest wall pain, five (31.2%) reported mild pain, and one (6.2%) reported moderate pain. All patients remain disease free at last follow-up. The median ipsilateral breast, planning target volume (PTV), and implant volumes were 614, 57, and 333 cm^3^. The median ratios of PTV/ipsilateral breast volume (implant excluded) and PTV/total volume (implant included) were 9 and 6%.

**Conclusion:** These 16 breast cancer cases with prior bilateral augmentation treated with EB-APBI demonstrate favorable clinical outcomes. Further exploration of EB-APBI as a treatment option for this patient population is warranted.

## Introduction

The number of breast augmentation procedures in the United States has increased by 35% over the previous 12 years to 286,274 in 2012 (1) and remains one of the most common cosmetic procedures. It is the second leading cosmetic surgical procedure and the leading cosmetic surgery in women. Ninety percent of these procedures are performed in women <50 years of age.

Reports on outcomes of whole breast irradiation in women with previous augmentation (2–15) have described radiotherapy (RT)-induced circumferential fibrosis around the implant, leading to capsular contraction or other complications in as high as 65% of patients and satisfactory cosmesis in as low as 1/3 of the patients.

Accelerated partial breast irradiation (APBI) is currently under study as a treatment option for early stage [T*is*, T1, T2 (≤3 cm) N0 M0] breast cancer, with results pending from NSABP B-39/RTOG 0413 (16) and several other large randomized clinical trials. Theoretically, APBI would decrease the implant surface area exposed to radiation and thereby reduce the risk of capsular contracture and unsatisfactory cosmesis. However, there is limited data on the use of APBI in women who have had prior breast augmentation in the affected breast (17–21). The largest data series to date of APBI-treated augmented breasts utilized various brachytherapy techniques (17). External beam APBI (EB-APBI) using either 3-dimensional conformal radiotherapy (3D-CRT) or intensity-modulated radiotherapy (IMRT) is increasingly being investigated (16, 22, 23), but women with breast augmentation in the affected breast were excluded from B-39 (16), and many questions regarding the feasibility of external beam APBI in this population remain unanswered: Would APBI using 3D-CRT/IMRT planning cause complications specific to augmented breasts? Does this technique result in acceptable cosmetic outcomes? Does the cosmetic outcome change over time? This report presents our institutional experience to date of EB-APBI in the setting of previous breast augmentation.

## Materials and Methods

Between April 2008 and November 2012, 16 newly diagnosed stage 0 or 1 breast cancer patients with prior breast augmentation were treated on one of two IRB-approved institutional post-lumpectomy EB-APBI studies: Phase II APBI utilizing IMRT (*n* = 3) or Phase III APBI randomized IMRT vs. 3D-CRT (*n* = 13); both protocols utilized image-guided radiotherapy (IGRT). RT planning and treatment details have been previously reported (19, 23–25) and summarized here. RT planning included an initial computed tomographic (CT) simulation scan to ascertain the location and size of the surgical cavity, which was designated as the gross target volume (GTV). An additional margin of 1 cm was contoured around the GTV to form the clinical target volume (CTV). The planning target volume (PTV) was formed by adding an additional 0.5 cm around the CTV. The ipsilateral breast volume was contoured from the clavicle to the inframammary fold in the cranial–caudal direction, and medially/laterally from mid-sternum to approximately the mid-axillary line. The CTV and PTV volumes were at least 0.5 cm from the skin surface and were only allowed to extend into the implant volume for 0.5 cm. CTV and PTV were also at least 0.5 cm from the chest wall which included the ribs, intercostal muscles, and pectoralis muscles between the breast tissue and the lung/chest wall interface and was contoured from the mid-sternum medially to the level of the latissimus dorsi laterally. Ribs and muscular chest wall could be excluded from both the PTV and CTV at the discretion of the treating physician. Finally, a wire was placed on the skin surface to indicate the surgical scar to possibly aide in target definition.

Dose constraints for IMRT patients were as follows: The PTV/ipsilateral breast volume ratio was generally limited to <20%. Plans were optimized so that ≥95% of the PTV received ≥95% of the prescribed dose. Heart exposure was limited to ≤5% organ volume receiving >5% of the prescribed dose. Ipsilateral lung exposure was initially limited to ≤15% receiving 30% of the prescribed dose, then reduced to ≤10% receiving >30% of the prescribed dose, and eventually further reduced to ≤10% receiving >20% of the prescribed dose for the remaining cases in this series after we gained more experience with IGRT. There were no dose constraints for the breast implant, since its composition was considered to be inert. Sub-groups of dose–volume data were compared using the two-sample *t* test after verifying normality and compensating for variance.

In both APBI protocols, breast/chest wall pain and cosmesis were rated by patient at each follow-up. The Phase III protocol additionally included a pain and cosmesis evaluation at baseline (post-lumpectomy pre-RT). Patients on the Phase II protocol were asked to verbally rate pain as none, mild, moderate, or severe, and cosmesis as excellent, good, fair, or poor without further instructions. Patients on the Phase III protocol completed questionnaires. Possible responses for pain were no pain, mild tenderness or infrequent discomfort, mild frequent pain, moderate and constant pain, severe pain, and pain requiring hospitalization. Choices for overall appearance and specifically size, shape/contour, texture/firmness, and skin/color, were no change or minimal change, slightly different, obviously different, and drastically different. Cosmesis was also evaluated by physician at each follow-up (and at baseline on the Phase III protocol) per RTOG criteria as detailed in RTOG 0413/NSABP B-39 (16). Presumed surgical effects on cosmesis were not excluded. The National Cancer Institute Common Terminology Criteria for Adverse Events version 3.0 (CTCAE v3.0) was used to grade toxicities.

## Results

### Patient and treatment-related characteristics

Sixteen patients treated with EB-APBI in the setting of previous breast augmentation were evaluated. The median follow-up from APBI completion was 23.9 months (range, 1.2–58.6). Patient characteristics (see Table 1) included median age of 50.7 years (range, 40.6–62.2) and 9/16 (56.2%) postmenopausal at diagnosis, median tumor span of 0.75 cm, and 10/16 (62.5%) with final margins of at least 0.5 cm. The median time from augmentation mammoplasty to breast cancer diagnosis was 10.7 years. The majority of the implants were saline prostheses (*n* = 13) placed subpectorally (*n* = 15). All patients tolerated treatment well and completed a total dose of 38.5 Gy in 10 equal fractions delivered twice daily over five consecutive days. Fifteen patients were treated with systemic therapy after APBI completion. All patients remain disease free at last follow-up.

**Table 1:** **Patient characteristics**.

Age at diagnosis (year)	40–49					7
	50–59					5
	≥60					4

Menopausal status (*n*)	Pre/perimenopausal					7
	Postmenopausal					9

Histology/grade (*n*)	DCIS		6	Low		3
				Intermediate		3
				High		0
	Invasive	T1a	2	Low		6
		T1b	6	Intermediate		3
		T1c	2	High		1

Tumor span (cm)	Median (range)			0.75 (0.2–3.0^a^)		

Margin (cm)	Median (range)			0.5 (0.2–1.5)		

Breast laterality/quadrant (n)	Right	UO	5	Left	UO	4
		UI	2		UI	1
		LO	0		LO	2
		LI	1		LI	1

Position of implant (*n*)	Retroglandular					1
	Subpectoral					15

Type of implant (*n*)	Saline					13
	Silicon					3

Time from augmentation to RT (year)	Median (range)			10.7 (2.3–20.2)		
Systemic therapy post-RT (*n*)	Chemotherapy only					1
	Chemotherapy and endocrine therapy					2
	Endocrine therapy only					12
	None					1

*DCIS, ductal carcinoma *in situ*; UO, upper outer; UI, upper inner; LO, lower outer; LI, lower inner; RT, radiotherapy*.

*^a^ All invasive tumors were ≤2.0 cm in size. One of the DCIS tumors was 3.0 cm in maximum dimension*.

### Dose–volume analyses

Five cases in this series were treated with 3D-CRT, while the remaining 11 were treated with IMRT. Table 2 summarizes the dose–volume data from these 16 cases. The median PTV was 57 cm^3^ (range, 34–248). The median ipsilateral breast tissue (including the PTV and excluding the implant) volume was 614 cm^3^ (range, 269–2073). The median total (breast tissue plus implant) volume was 997 cm^3^. The median PTV/ipsilateral breast volume and PTV/total volume ratios were 9 and 6%, respectively.

**Table 2:** **Dosimetric analysis**.

Structure	*V* % _isodose_	Median (range) or *n*	
Ipsilateral breast	105	0.5% (0–12)	
	100	6% (2–15)	
	75	13% (8–23)	
	50	20% (12–31)	
	25	31% (19–46)	
Breast/implant interface^a^	100	1% (0–27)	
	75	7% (0–34)	
	50	12% (0–40)	
	25	22% (0–71)	
GTV	95	100% (95–100)	
	90	100% (99–100)	
CTV	95	100% (95–100)	
	90	100% (99–100)	
PTV	95	96% (81–100)	
	90	100% (95–100)	
PTV (*n*)		<50 cm^3^	7
		50–100 cm^3^	8
		>100 cm^3^	1
Breast volume (*n*)		<500 cm^3^	7
		500–1000 cm^3^	8
		>1000 cm^3^	1
PTV/breast volume		9% (6–14)	
Implant volume		344 cm^3^ (248–527)	
PTV/implant volume		17% (7–61)	
Total volume^b^		997 cm^3^ (624–2482)	
PTV/total volume		6% (3–10)	
Implant/total volume		36% (16–57)	

**V*%_isodose_, volume (%) of organ/structure receiving a specific % of the prescribed radiation dose; GTV, gross treatment volume, corresponding to the lumpectomy cavity; CTV, clinical treatment volume; PTV, planning treatment volume*.

*^a^ A breast/implant interface volume was created by contouring any breast or implant volume within 5 mm of the actual breast/implant interface*.

*^b^ Total volume = combined ipsilateral breast and implant volume*.

**Figure 1:**
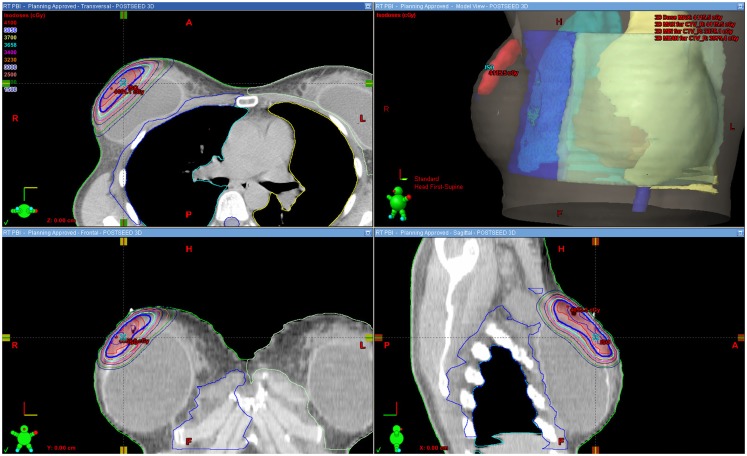
**Sample approved treatment plan**.

Figure 1 shows an example of an approved treatment plan. Only a small portion of the ipsilateral breast received high percentages of the prescribed dose. Table 2 shows dose–volume data from the 16 treatment plans. The median percentages receiving 100, 75, 50, and 25% of the prescribed dose (V100, V75, V50, and V25) to the ipsilateral breast were 6, 13, 20, and 31%, respectively. The median V105 to the ipsilateral breast was 0.5% (range, 0–12) and 4.2 cm^3^ (range, 0–78.3); there was no statistically significant difference between the V105 values (*p* = 0.229 for percentage volume and *p* = 0.147 for cubic centimeter) for patients with ipsilateral breast volume <500 cm^3^ (*n* = 7) vs. >500 cm^3^ (*n* = 9). V110 to the ipsilateral breast was noted as 0.00016% in 1/16 plans and negligible in all other cases. The median V105 to the total volume (combined breast tissue and implant) was 0.5% (range, 0–10) and 5.5 cm^3^ (range, 0–113.4). The median V100, V75, V50, and V25 to the breast-implant interface volume were 1, 7, 12, and 22%, respectively. The median prescription isodose coverage to 95 and 90% of the GTV, CTV, and PTV structures were high at 100, 100, 100 and 100, 96, 100%, respectively.

### Cosmesis reported by patient and physician

**Figure 2:**
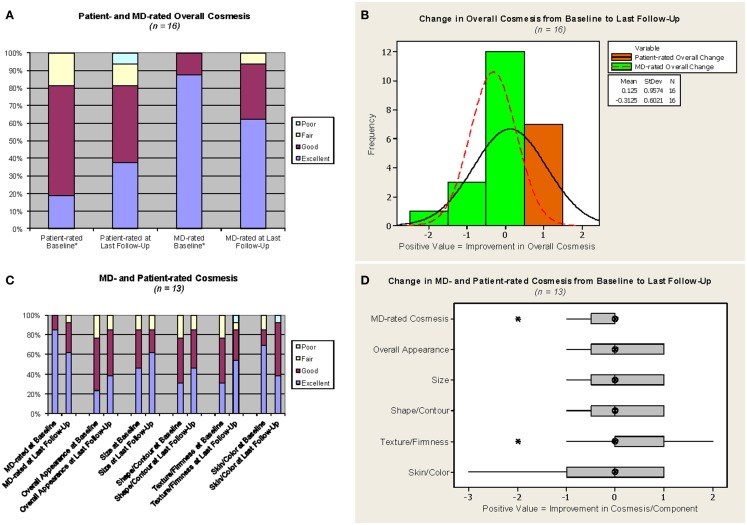
**Cosmesis outcomes**. MD = study investigator. *For the three cases on the Phase II protocol, which did not require a baseline assessment, values from the 1-month assessment were used as proxy for baseline. Phase III protocol questionnaire responses were converted as follows: no change or minimal change = excellent, slightly different = good, obviously different = fair, drastically different = poor. The median follow-up from APBI completion for **(C,D)** was 23.4 months. **(A,C)** Light blue = poor, yellow = fair, maroon = good, purple = excellent. **(B)** Orange with solid line = patient-rated overall change, green with dotted line = MD-rated overall change. **(D)** Hatched circle = median, shaded box = interquartile range, asterisk = outlier (more than 1.5 x interquartile range below the first quartile or above the third quartile).

**Figure 3:**
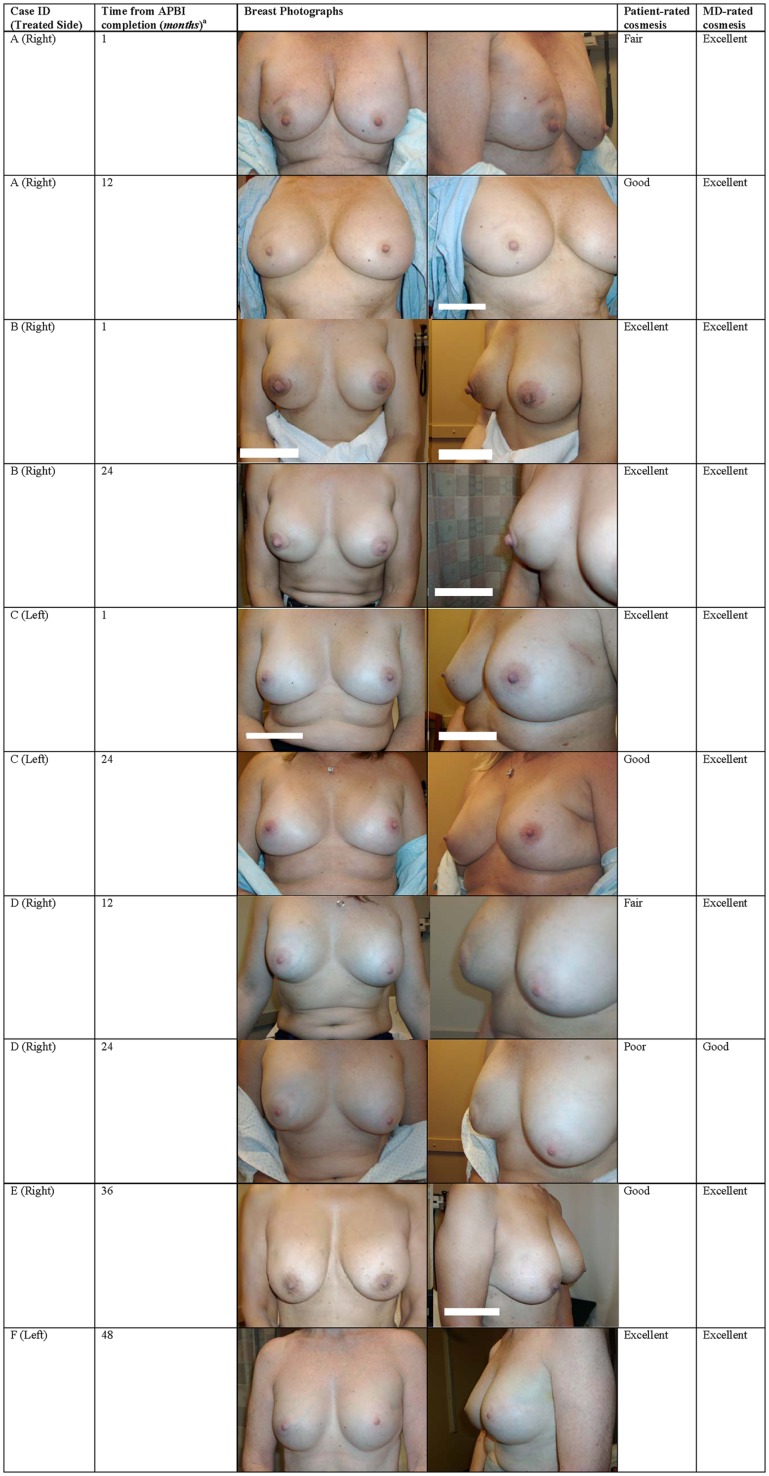
**Breast photographs and cosmesis after EB-APBI**. ^a^Time from APBI completion >1 month designated as the closest 6 months follow-up time point.

Cosmetic outcomes analyzed both in aggregate and at the individual case level are represented pictorially in Figure 2 and summarized in Table 3. Figure 3 shows representative digital breast photographs taken at ≥12 months after APBI completion; photos taken at 1 month after APBI were additionally included for comparison when available. Overall cosmesis was judged by patient at baseline (Phase III protocol, *n* = 13) or at 1 month following APBI completion (Phase II protocol, *n* = 3) as excellent: 3/16, good: 10/16, and fair: 3/16; physician assessment at the same time points indicated excellent: 14/16, and good: 2/16. Cosmesis at last follow-up was judged by patient as excellent: 6/16, good: 7/16, fair: 2/16, and poor: 1/16; physician assessment indicated excellent: 10/16, good: 5/16, and fair: 1/16, with minimal change in overall cosmesis from baseline to last follow-up within individual cases, see Figure 2B. There was a high degree of agreement between the physician- and patient-rated cosmesis at last-follow-up, with 14/16 having the same rating or varying by one gradation (e.g., excellent vs. good).

**Table 3:** **Cosmesis at specific time points**.

Visit^a^	Patient-rated cosmesis		Physician-rated cosmesis	
	*n*^b^	Excellent/good	*n*^b^	Excellent/good
Baseline	16	13 (81.2%)	16	16 (100%)
12 months	15	13 (86.7%)	15	15 (100%)
24 months	11	8 (72.7%)	11	11 (100%)
36 months	3	3 (100%)	3	3 (100%)
48 months	3	3 (100%)	3	3 (100%)
60 months	1	1 (100%)	1	1 (100%)

*^a^ From APBI completion, closest follow-up within ±180 days to time point specified*.

*^b^ Breasts with evaluated cosmesis/pain at time point specified*.

The stability between cosmesis pre-RT and at last follow-up is confirmed when only cases with pre-RT assessments are considered. The aggregate excellent/good overall cosmesis as well as component category results generally remained stable or improved for the 13 Phase III patients, see Figure 2C. Similarly, comparison between individual case level cosmesis at baseline and last follow-up showed little or no change, see Figure 2D. One patient (case D in Figure 3) reported a decline of cosmesis by more than one gradation (e.g., from good at baseline to poor at last follow-up); however, the treating physician judged her cosmesis as good at the same time point.

### Treatment-related toxicities

No capsular contracture, implant rupture, or any other implant-related complications were observed. No edema or telangiectasia was reported at last follow-up. Approximately 12 weeks after APBI completion, one of the patients treated with 3D-CRT developed a painful red and swollen breast with a persistent seroma treated empirically with antibiotics, oxycodone for pain, simple aspiration, and ultimately incision and drainage. There was no evidence of infection by microbiological culture or imaging. Symptoms were resolved by the next follow-up at ~1 month after last drainage. Another patient treated with 3D-CRT – who had the highest V105 and the only measurable V110 in these 16 cases – developed grade 2 subcutaneous fibrosis about 6 months after APBI completion; according to the patient, the pain is “quite manageable with half a pill of [hydrocodone 5 mg/acetaminophen 300 mg]” as needed, which she has taken ever since her lumpectomy and also uses it to manage tamoxifen-related breast tenderness. Of note, this particular patient continued to judge her cosmetic outcome as good at last follow-up, noting that the increased thickening has only slightly altered the contour and firmness of her treated breast, while her treating physician downgraded her cosmesis to fair due to the increased thickening. No other acute or chronic grade 2+ adverse events, other uses of antibiotics, or pain requiring narcotics were observed.

**Figure 4:**
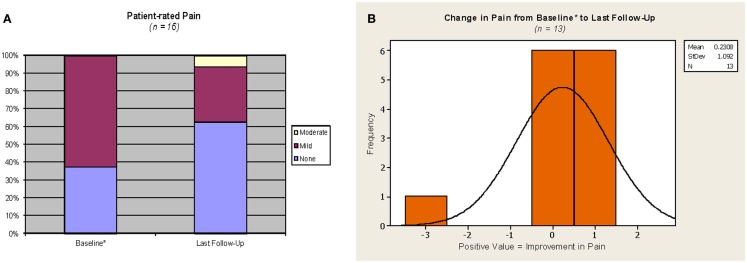
**Pain outcomes**. *For the three cases on the phase 2 protocol, which did not require a baseline assessment, values from the 1 month assessment were used as proxy for baseline. Phase III protocol questionnaire responses were converted as follows: no pain = none, mild tenderness or infrequent discomfort or mild frequent pain = mild; moderate and constant pain = moderate; severe pain or pain requiring hospitalization = severe. **(A)** Yellow = moderate, maroon = mild, purple = none. **(B)** Orange with solid line = patient-rated change in pain.

Patient-reported breast/chest wall pain outcomes are represented in Figure 4. Pain was judged by patient questionnaire at baseline (*n* = 13) or verbal rating at 1 month following APBI completion (*n* = 3) as none: 6/16, and mild: 10/16. Pain at last follow-up was judged by patient as none: 10/16, mild: 5/16, and moderate: 1/16 (corresponding to the aforementioned patient with grade 2 fibrosis). There was no change in patient-rated pain from baseline to last follow-up in 8/16 cases. Of the eight cases with a change in pain level, 6/8 reported an improvement (i.e., a decrease in pain), and the two remaining patients reported a change from no pain at baseline to mild (1/16) and moderate (1/16) pain at last follow-up. The change in pain from baseline to last follow-up at the individual case level was small among the 13 Phase III patients, see Figure 4B.

## Discussion

Since breast-conserving treatment with RT became an accepted alternative to mastectomy, some investigators have reported on the use of whole breast irradiation in the setting of previous augmentation (2, 4–15). The implications of reported disease control and cosmetic outcomes have sometimes been obscured due to heterogeneous populations that have included previously augmented breasts treated with surgery only and no radiation (13, 14), breasts reconstructed after mastectomy (4, 6, 7, 12), and a wide array of clinical scenarios including positive surgical margins (9), locally advanced (10, 11), recurrent (4), and metastatic (5) disease. These publications have described ~55% rate of painful hardening of the implants after RT, with good/excellent cosmesis ranging from as low as 1/3 (5) to as high as 100% (6, 12, 15).

The data on the use of APBI in women with prior breast augmentation in the affected breast is even more limited (17–20). Our previous publication (19) is the only investigation utilizing external beam rather than brachytherapy, and the only full-length treatment apart from a few case reports (18, 20). Findings from these reports of whole breast or partial breast irradiation are summarized in the Table S1 in Supplementary Material.

Possible explanations for unsatisfactory cosmesis put forth by these studies include surgical technique used in the augmentation mammoplasty procedure (2, 4), time from mammoplasty to RT (4, 6), implant location (retroglandular vs. subpectoral) (5, 9–11, 14), age (6), dose (4, 10), use of bolus or other practices resulting in dose inhomogeneity (6, 12), use or timing of chemotherapy (9, 11), etc., with little consensus on the impact of any of these factors on clinical outcomes.

Combining the data presented in this report with previously reported outcomes from primarily stage 1–2 breast cancer in patients with previous augmentation (2–10, 12, 14, 15, 17, 18, 20) demonstrate excellent disease control after both whole breast irradiation and APBI. After 0.5 to over 10 years of follow-up, cases treated with adjuvant RT generated a crude local failure rate of 2.3% (5/221) and overall disease free survival rate of 95.5% (211/221). Cases treated with APBI had a crude local failure of 1.7% (2/120) with no distant failures (17, 18, 20, 21). Although longer follow-up is necessary, these efficacy rates are certainly comparable to rates reported after whole breast irradiation and APBI in early stage breast cancer in patients without prior breast augmentation. These favorable results support the consideration of breast-conserving surgery followed by RT, and specifically APBI, in the setting of previous breast augmentation in early stage breast cancer patients deemed otherwise suitable for APBI.

Substantial advances in RT planning and delivery have been made since the initial reports of whole breast irradiation in patients with prior breast augmentation. The earliest publications (2–6) included patients treated before the widespread adoption of CT-based planning with digitally reconstructed radiographs. While planning details are not described in the reports of whole breast irradiation, the higher rate of excellent/good cosmesis in the more recent publications may reflect the increasing usage of more sophisticated planning and treatment techniques. Every APBI publication reported utilizing CT-based planning and IGRT (17–20) to optimize conformality to the PTV while minimizing hot or cold spots as well as overall dose to implant prostheses. Combining our data with other APBI cases in previously augmented breasts (17, 18, 20) yields an overall rate of 79.2% excellent cosmesis and 95.8% excellent/good cosmesis. Only 5% of all APBI cases collectively reported in the current literature developed any new capsular contracture after RT and none required surgical revision, scar release, or implant removal (17, 18, 20).

Recent reports of inferior cosmetic outcomes after EB-APBI (in patients without prior augmentation) have attracted much attention. Adverse cosmesis and toxicities were initially noted by groups from Tufts and University of Michigan (26, 27). The large randomized Canadian RAPID trial then released interim results showing that APBI delivered with 3D-CRT was associated with worse cosmetic outcomes and late radiation changes at 3 years compared to whole breast irradiation (28). Liss et al. from University of Michigan recently provided an update showing further decline in cosmetic outcome between 2.5 and 5 years of follow-up (29). Our previous report (30) offered an extensive discussion of the discrepant cosmesis reported by various EB-APBI studies (30) in relation to subtle but important differences in treatment parameters, including our use of IGRT utilizing non-migrating fiducials and the use of mandatory respiratory control in the University of Michigan trial, as well as lower mean V100 and V50 (30) in our treatment plans. In their discussion, Liss et al. interpreted our previously published data (30) as also showing a trend toward decline in cosmesis over time (29). At first glance, their 5-year outcomes and interpretation of our previously published data seem to undercut the longer-term significance of the currently favorable cosmetic outcomes presented in this analysis. It is worth noting, however, that the 41 cases evaluable at 5 years in our previous report retained a much higher rate of excellent/good cosmesis compared to the 34 cases from the University of Michigan (87.8 vs. 73.3%). Therefore, even if a continued decline over time is observed in our EB-APBI studies with longer follow-up, the cosmetic outcomes – including those in patients with prior augmentation – will likely remain acceptably favorable. Furthermore, and perhaps more importantly, since whole breast irradiation has historically demonstrated high rates of capsular contracture and unsatisfactory cosmesis, APBI, whether delivered with external beam or brachytherapy, may provide a more palatable alternative to patients with prior augmentation who prefer breast-conserving therapy.

In summary, our institutional experience noted no RT-induced capsular contracture, implant-related fibrosis or complication, or high rates of unsatisfactory cosmesis previously observed in some whole breast irradiation studies. We previously demonstrated the feasibility of using external beam to deliver APBI in cosmetically augmented breasts (19). EB-APBI is non-invasive and offers the advantages of decreased procedural trauma to the breast and relative ease of adoption for most radiation treatment facilities, thus potentially expanding the accessibility of APBI to more patients with prior augmentation mammoplasty. The precision required to optimize dose homogeneity and customize implant-sparing is particularly amenable to manipulation with IMRT (23–25, 31). This updated and expanded data series corroborates and strengthens our previous report that breast-conserving therapy including APBI in general, and EB-APBI in particular, is a viable treatment option for patients with prior breast augmentation.

## Conflict of Interest Statement

The authors declare that the research was conducted in the absence of any commercial or financial relationships that could be construed as a potential conflict of interest.

## Supplementary Material

The Supplementary Material for this article can be found online at http://www.frontiersin.org/Journal/10.3389/fonc.2014.00154/abstract

Table S1 **Reports of breast irradiation after breast augmentation or reconstruction**.
