# Supplementary material for: External Beam Accelerated Partial Breast Irradiation Yields Favorable Outcomes in Patients with Prior Breast Augmentation
Source: Front Oncol. 2014 Jun 19;4:154. doi: 10.3389/fonc.2014.00154 (PMC4062870; doi:10.3389/fonc.2014.00154)
Supplement: Table S1 — Reports of breast irradiation after breast augmentation or reconstruction. [file Data_Sheet1.DOC]

Supplemental Table. Reports of breast irradiation after breast augmentation or reconstruction

| **First author and year of publication** | **Sample Characteristics (*n*)** | **Implant to RT interval** | **Follow-Up (year)** | **RT modality** | **Fractionation scheme** | **Disease Recurrence (*n*)** | **Cosmesis** | **Capsular Contraction/Implant Encapsulation** | **Toxicity (all grades)** |
| --- | --- | --- | --- | --- | --- | --- | --- | --- | --- |
| Jacobson 1986 (2) | 3 augmented breasts (all submuscular silicone) | 1-3 months (cancer incidentally discovered at augmentation) | ~1-3.6, *NOS* | External beam with tangential fields | ~5000-6000 rads to whole breast/5 weeks | None | Excellent: 1; Good: 1; Poor:1 | *NR* | 1/3 wound breakdown, prosthesis sloughed |
| Lafreniere 1987 (3) | 1 (age 39; tumor size = 1 cm; retroglandular; silicone) | 5 years | 1.2 | External beam with tangential fields | 5000 rads to whole breast; 900 rads boost | None | *NR* | *NR* | *NR* |
| Halpern 1990 (4) | 6 augmented breasts (all subcutaneous); overall 13 breasts (T1N0 = 4, T1N1 = 2, T2N0 = 3, T2N1 = 1, T2N2 = 1) | Augmented breasts: mean 5.6 years (range, 3-8); 3 other cases underwent mastectomy & reconstruction < 5 months prior to RT | (*n* = 11) Median 6.5 (range, 3-16) | External beam with tangential fields | 45-50 Gy to whole breast/5 weeks; 10-21 Gy boost | Overall: No local failures, 2 cases with distant metastases | Overall: Excellent: 0; Good: 3; Fair: 1; Moderate: 2; Poor: 5 | Overall: Fair (mild fibrosis and capsular contraction of implant with minimal shrinkage) = 1; Moderate (shrinkage of breast to < 2/3 initial size) = 2; Poor (severe fibrosis and contracted breast) = 5 | *NR* |
| Ryu 1990 (5) | 3 augmented breasts (case #1: T1N1, subcutaneous silicone prosthesis; #2: T2N1, subcutaneous silicone prostheses; #3: TxN2M1, injected silicone) | #1: 13 years; #2: 5 years; #3: 28 years | #1: 5 years; #2: 2.5 years; #3: 2.5 years | External beam with tangential fields | #1: 45 Gy to upper 1/2 breast,, 14 Gy axilla boost; #2: 46.8 Gy to whole breast, 16 Gy boost, #3: 62 Gy to whole breast, 5 Gy boost to axilla and supracalvicular fossa, 5 Gy boost to internal mammary node | None (case #3 treated with primary radiation therapy alone, no surgery, alive with disease) | Excellent: 0; Good: 1; Fair: 2 (case #2 had subsequent scar release procedure and attained excellent cosmesis with procedure) | 1 (case #2) | #3 Moist desquamation requiring 4 week treatment break, also late fibrosis |
| Kuske 1991 (6) | 5 augmented breasts; overall 72 breasts, 66 patients (T*is* = 2, T1 = 20, T2 = 28; T3 = 13, T4 = 7; N0 = 31, N1 = 33, N2 = 5, N3 = 1) | *NR* | Overall: median 4 (range, 1-14) | External beam with tangential fields followed by electron boost | 50.4 Gy to whole breast/21 fractions; 10 Gy/5 fractions boost | Augmented cases: None | *MD-rated:* Excellent/Good: augmented breasts 5/5, overall 34/69 (49%); *Patient-rated:* Excellent/ Good: overall 28/42 (65%) | Overall: slightly thickened, slight deformation = 38%; firm to hard, moderate deformation = 13%; hard, severe deformation = 13% | Augmented breasts: 1/5 moderate fibrosis |
| Chu 1992 (7) | 7 augmented breasts (Stage 0 = 1, Stage I = 1, Stage II = 5); overall 39 breasts | Augmented breasts: 5-17 years | Augmented breasts: mean 3.6 (range, 2.9-7) | External beam with tangential fields followed by photon or electron boost | 48 Gy to whole breast/5 weeks; 14 Gy boost/1.5 weeks | Augmented breasts: No local failures, 1 case of distant metastases | Augmented breasts (*n* = 7): Excellent: 4, Good: 2, Fair: 1; Overall (*n* = 39): Excellent/ Good: 34 (87%) | Augmented breasts: moderate skin tanning, moderate fibrosis and capsular contraction, but no serious distortion = 1; *NOS* | Overall: 31% grade 2+ complications |
| Krishnan 1993 (8) | 5 augmented breast (T1N0 = 3; T1N1 = 2) | 8-16 years | *(From diagnosis)* Median 5 (range, 2-7) | Interstitial brachytherapy with Ir-192 followed by external beam | 15-20 Gy perioperative boost to tumor bed; 45 Gy to whole breast | No local failures | Excellent: 2; Good: 2; Fair: 1 | *NR* | *NR* |
| Guenther 1994 (9) | 20 augmented breasts (mean age at diagnosis 52, range 34-72; positive margins = 3, mean tumor size = 1.43 cm, 75% T1, node positive = 5; subcutaneous = 14, retromuscular = 6) | *NR* | Median 3.8 (range, 0.5-9.3) | External beam with tangential fields followed by photon, electron, or Ir-192 implantation boost | 45-50 Gy to whole breast; 13-21 Gy boost | No local failures; 2 cases of distant metastases | Excellent: 11; Good: 6; Fair: 2; Poor: 1 | 1 case of ruptured implant with extensive fibrosis leading to subsequent surgical revision with capsulectomy and bilateral implant removal; *NOS* | Late skin and subcutaneous tissue toxicity: 20% grade 2+ |
| Mark 1996 (10) | 21 augmented breasts (median age at diagnosis 50, range 36-70; T*is* = 2, T1N0 = 8; T1N1 = 6, T2N0 = 2, T2N1 = 2, T3N1 = 1; subcutaneous = 13, retromuscular = 8) | Median 7 years (range, 0.4-28) | Median 1.8 (range, 0.3-4) | External beam with tangential fields followed by electron boost | 45-56 Gy to whole breast/25-28 fractions; 10-20 Gy boost | 1 local failure, 2 cases of distant metastases | Excellent: 7; Good: 2; Fair: 2; Poor:10 | 12 (57%), 7 attempted surgical repair | *NR* |
| Handel 1996 (11) | 26 augmented breasts (mean age at diagnosis 45, range 31-67; mean tumor size 1.78 cm, range 0.8-5.0; node positive = 8; submuscular = 6, subglandular = 20) | Mean 7.5 years (range, 0.5-20) | *NR* (mean time to contracture 22.4 weeks, range 7.6-39.7; mean follow-up for cases without contracture = 158 weeks) | External beam with tangential fields followed by photon, electron, or Ir-192 implantation boost | 45-56 Gy to whole breast/23-26 fractions; 5.4-25.3 Gy boost | 2 dead with disease, *NOS* | Excellent: 7; Good: 7; Fair/Poor:12^a^ | 17 (65%), 8 have undergone surgical correction | *NR* |
| Victor 1998 (12) | 8 augmented breasts (Stage 0 = 1, Stage I = 4, Stage II = 3; submuscular = 5, subglandular = 3); 13 reconstructed (Stage IIA = 2, Stage IIB = 6, Stage IIIA = 4, Stage IIIB = 1) | Augmented breasts: 0.4-21 years | Surviving patients (n = 17) median 2.7 (range, 0.4-6.9) | External beam with tangential fields followed by electron boost | Median doses of 50 Gy to whole breast; 10 Gy boost | Augmented breasts: 2 local failures | Augmented breasts: Excellent/Good: 8/8 ^b^ | Augmented breasts: none (*n* = 8) | No soft tissue necrosis, hematomas, severe pain, arm edema, brachial plexopathy, rib fractures, prosthetic leakage, frozen shoulders, or systemic symptoms; *NOS* |
| Karanas 2003 (13) | 19 augmented breasts received RT; overall 28 augmented breasts initially treated with BCT | *NR* | Overall: mean 3.2 (range, 0.25-10.2) | *NR* | RT to whole breast, *NOS* | Overall: 7/28 local failures | Poor: 1/19; *NOS* | 3/19 cases of capsular contraction; 3/19 cases required implant removal and/or revision | 11/19 experienced implant-related complications as follows: implant infection: 2; intractable pain: 2; poor cosmesis: 1; capsular contraction: 3; erosion: 1; intractable seroma: 1; rupture: 1 |
| Gray 2004 (14) | 17 augmented breasts treated with BCT including RT (subpectoral = 9, subglandular = 8); overall 40 augmented breasts (median age at diagnosis 50.7, range 36-65; median tumor size = 1.2 cm, range 0.3-4.0) | Overall: median 7 years (range, 1-20) | Overall: median 3 (range, 0.2-6.1) | External beam with tangential fields followed by electron boost | 48.6 Gy/27 fractions to whole breast; 12 Gy boost/6 fractions | Augmented breasts treated with RT (*n* = 17): No local failures | Excellent/Good: 11/17 (64.7%); Fair/Poor: 6/17 (35.3%) ^c^ | 5/17, 3 requiring implant removal or capsuletomy with implant exchange | *NR* |
| Tuli 2006 (15) | 6 augmented breasts treated with BCT including RT (Stage I = 2, Stage II = 4); overall 12 augmented breasts (average age 49.2, range 31-63; subglandular = 4, subpectoral = 8) | Overall: average 12.6 years (range, 1-31) | Overall: average 3.8 (range, 0.7-10.2) | External beam with tangential fields | 45-50.4 Gy/27 fractions; 12 Gy boost | Augmented breasts treated with RT (*n* = 6): None | 6/6 maintained favorable cosmesis, *NOS* | None (*n* = 6) | No implant- or irradiation-related complications (*n* = 6), *NOS* |
| Bloom 2011 (18) | 1 (age 55; intermediate grade DCIS; subpectoral saline) | 8 years | 0.5 | Brachytherapy using SAVI with Ir-192 | 34 Gy in 10 BID fractions | None | Excellent | Patient had capsular contracture and fibrosis, which was more noticeable in the left breast, predating diagnosis; patient did undergo right breast lift for symmetry | Hyperpigmentation grade 1 at 8 weeks |
| Dragun 2011 (20) | 1 (age 51; intermediate grade DCIS; subpectoral saline) | ~8 years | 1 | Brachytherapy using MammoSite ML catheter with Ir-192 | 34 Gy in 10 BID fractions | None | Excellent | None | Grade 2 radiation dermatitis at 7-10 days post-RT, resolved within 6 weeks post-RT; no long-term skin toxicity or significant evidence of RT-induced pain, hyperpigmentation, telangiectasia, or fibrosis |
| Kuske 2012 (17, 21) | 102 (median age 52; ; Tis = 21, T1 = 74, T2 =7, N1 = 8; retroglandular = 23, subpectoral = 76, unknown = 3) | *NR* | 3 (range, 1-8.3) | Brachytherapy using with interstitial implant (n = 91), SAVI (n = 8), SAVI + flanking catheters (n =1), or Contura (n = 2) with Ir-192 | 34 Gy in 10 BID fractions | 2 cases of local/regional failures | Excellent: 80%; Good: 15%; Fair 4%; Poor 1% | 11% (5% with baseline contracture unchanged with RT); no surgical intervention needed | No implant rupture; erythema 43/102 (42.2%, 4 cases with fever), antibiotic use 38/102 (37.3%, 11 prophylactic); chronic pain grade 1: 6%; telangiectasia grade 1: 8%; no fat or skin necrosis; fibrosis: 27% (1% grade 2); 1 case of wound dehiscence; 1 case of pneumothorax; 1 case of persistent seroma |
| Current study | 16 (median age 50.7, range 40.6-62.2; Tis = 6, T1N0 = 10; retroglandular = 1, subpectoral = 15; saline = 13, silicone = 3) | Median 10.7 years (range, 2.3-20.2) | 1.5 (range, 0.3-4.9) | External beam with either 3D-CRT or IMRT | 38.5 Gy in 10 BID fractions | None | *MD-rated:* Excellent: 13; Good: 3; *Patient-rated:* Excellent: 7; Good: 8; Fair: 0; Poor: 1 | None | No implant rupture; no increase in patient-reported pain from baseline and no edema or telangiectasia at last follow-up; 1 case of grade 3 seroma, no other grade 2+ AE |

Abbreviations: RT = radiotherapy; *NOS* = not otherwise specified; *NR* = not reported; BID = twice daily; BCT = breast conserving therapy; DCIS = ductal carcinoma *in situ*; SAVI = strut-adjusted volume implant; 3D-CRT = three-dimensional conformal radiotherapy; IMRT = intensity-modulated radiotherapy

^a^ Cosmesis reported per authors’ own conversion from Baker grading as described in the discussion section, i.e., excellent = no skin change, fibrosis, or shrinkage = Baker grade 1 or 2; good = minimal fibrosis, light retraction of implant = Baker grade 3; fair = moderate capsular contraction = Baker grade 4; poor = marked fibrosis, contraction, and/or distortion = Baker grade 4

^b^ Cosmesis reported per authors’ own conversion from Baker grading as described in the methods section, i.e., excellent = Baker Class I; good = Baker Class II; fair = Baker Class III; poor = Baker Class IV

^c^ Cosmesis reported in Baker grades and converted as follows: Baker grades 1 or 2 and good subjective appearance = favorable aesthetic results = Excellent/Good; Baker grades 3 or 4 = Fair/Poor
